# Manual of the Diseases of the Eye

**Published:** 1901-01

**Authors:** 


MANUAL OF THE DISEASES OF THE EYE. For students and general practitioners. With 243 original illustrations, including 12 colored figures. By Charles H. May, M. D., Chief of Clinics and Instructor in Ophthamology, Eye Department, College of Physicians and Surgeons, Medical Department, Columbia University, New York. Price, net, $2.00. Publishers, William Wood & Company, New York.

This is a book intended more particularly for the student and the general practitioner of medicine, though the specialist will find in it many valuable suggestions.

It is especially comprehensive in the fundamental facts of ophthalmology. The illustrations are original, simple and of a thoroughly practical nature. We feel sure the book will meet with a hearty approval at the hands of the profession, and the author is to be congratulated for his success in adding another valuable book to the doctor's library. W. A. H.

